# Renal Biomarkers in Companion Animals—A Review

**DOI:** 10.3390/ani15060818

**Published:** 2025-03-13

**Authors:** Ana Filipa Pereira, Catarina Jota Baptista, Ana Faustino-Rocha, Paula A. Oliveira, Ana Cláudia Coelho

**Affiliations:** 1 Centre for the Research and Technology of Agroenvironmental and Biological Sciences (CITAB), Inov4Agro, University of Trás-os-Montes e Alto Douro (UTAD), Quinta de Prados, 5001-801 Vila Real, Portugal; anafaustino.faustino@sapo.pt (A.F.-R.); pamo@utad.pt (P.A.O.); accoelho@utad.pt (A.C.C.); 2 Animal and Veterinary Research Centre (CECAV), Associated Laboratory for Animal and Veterinary Sciences (AL4AnimalS), University of Trás-os-Montes e Alto Douro (UTAD), 5001-801 Vila Real, Portugal; 3 Egas Moniz Center for Interdisciplinary Research (CiiEM), Egas Moniz School of Health & Science, 2829-511 Almada, Portugal; 4 Department of Zootechnics, School of Sciences and Technology (ECT), University of Évora, 7004-516 Évora, Portugal; 5 Comprehensive Health Research Centre (CHRC), 7004-516 Évora, Portugal; 6 Department of Veterinary Sciences, UTAD, 5001-801 Vila Real, Portugal

**Keywords:** biomarker, kidney failure, small animal, veterinary

## Abstract

Recent advancements in molecular biology have led to the discovery of potential biomarkers for the diagnosis of acute kidney disease (AKD) and chronic kidney disease (CKD). The use of multiple biomarkers in the diagnosis of kidney disease has the potential to enhance both specificity and sensitivity, enabling early detection and intervention that could ultimately reduce morbidity and mortality rates. This review provides an overview of studies on urine and blood biomarkers and examines their utility and significance in various clinical settings. Further and continuous research is needed to support the application of these biomarkers in clinical practice to facilitate early diagnosis, guidance for different interventions, and the monitoring of disease progression.

## Simple Summary

Recent advancements in molecular biology have identified potential biomarkers for diagnosing kidney diseases. They may improve the specificity and sensitivity of diagnoses, allowing early detection and intervention. This review discusses studies on urine and blood biomarkers, highlighting their importance in various clinical settings. Ongoing research is necessary to integrate these biomarkers into clinical practice for early diagnosis, intervention guidance, and the monitoring of disease progression.

## 1. Introduction

Kidney diseases are frequently diagnosed in dogs and cats, with a high prevalence of chronic kidney disease (CKD) in older animals. Studies have shown that CKD affects approximately 0.5–1.0% of dogs and 1.0–3.0% of cats, with a significantly higher occurrence in the geriatric population, reaching up to 80% in cats [1,2,3]. Poor prognosis is often associated with advanced stages of kidney disease [1].

Renal injury leads to a decrease in kidney function, characterized by a reduction in the glomerular filtration rate (GFR) [4,5], leading to proteinuria through two main mechanisms: (1) increased plasma protein in the filtrate and (2) impaired tubular resorption of filtered protein [3].

The gold standard method for monitoring GFR and identifying this reduction is through inulin or iohexol clearance, but this method is time-consuming, technically challenging, and, therefore, impractical [6]. Thus, indirect markers in urine or blood are commonly used to assess kidney function [7].

The International Renal Interest Society (IRIS) has established a grading system for renal disease in both cats and dogs. This system incorporates criteria for both acute kidney disease (AKD) and chronic kidney disease (CKD), helping the identification and classification of the clinical cases and helping clinicians finding the most suitable therapeutic intervention for each case [8]. The grading system is based on two main biomarkers: serum creatinine (sCR) and symmetric dimethylarginine (SDMA) concentrations. Additionally, it further classifies renal disease based on the presence of proteinuria and systemic arterial blood pressure [9].

Despite these advancements, renal disease mortality remains high, primarily due to delayed detection and insensitive diagnostic tests [10]. The biomarkers suggested by IRIS have been shown to be suboptimal for the early detection of renal diseases in this species [10]. Consequently, there is a growing interest and ongoing research aimed at identifying novel biomarkers that could facilitate the early prediction and diagnosis of kidney diseases [10,11]. This review aims to summarize the current knowledge regarding renal biomarkers in cats and dogs. Although numerous biomarkers have the potential for early diagnosis, this review focuses on a few that have been extensively reported in the literature.

## 2. General Aspects of Renal Disease Biomarkers

Renal disease biomarkers are substances present in urine or blood that can indicate the presence and severity of renal injury. They provide insights into the specific location of damage and the potential for recovery [12]. For a molecule to be considered a good renal biomarker, it must exhibit the characteristics summarized in Table 1.

**Figure 1:**
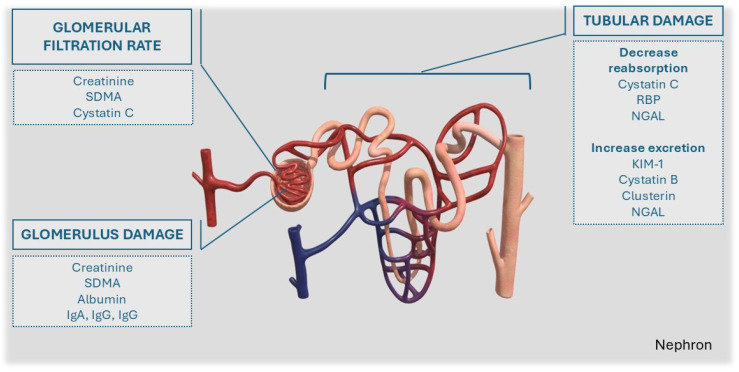
The distribution of renal biomarkers according to this anatomical site. (IgA) Immunoglobulins A, (IgM) Immunoglobulins, (IgG) Immunoglobulins G; (SDMA) Symmetric dimethylarginine; (NGAL) Neutrophil Gelatinase-Associated Lipocalin; (KIM-1) Kidney Injury Molecule-1; Retinol Binding Protein (RBP).

Renal biomarkers can be categorized into two main groups: markers of reduction in GFR and markers of renal damage. Inside this last category, two subcategories may be considered: markers of glomerular damage and markers of tubular dysfunction [14] (Figure 1). In general, the presence of high- and intermediate-molecular-weight proteins in the urine are indicative of glomerular damage, while low-molecular-weight proteins and enzymes suggest tubular damage. However, in practice, the complexity of kidney injuries and the concurrent involvement of other organs can alter the concentration of these molecules, potentially diminishing the diagnostic value of these markers.

## 3. Markers of Glomerular Filtration Rate (GFR)

### 3.1. Serum Creatinine (sCr)

sCr is a byproduct of a non-enzymatic process in which creatine and phosphocreatine, primarily stored in muscle tissue, are converted into creatinine [15]. Once in the bloodstream, creatinine is freely filtered by the glomeruli and is neither reabsorbed nor secreted by the renal tubules [3]. In cases of renal dysfunction, sCr levels typically increase, making it the most commonly used biomarker in veterinary medicine for assessing renal function. However, its use as a marker of GFR has been questioned due to the influence of various non-renal factors, such as dehydration, diet, breed, age, and body weight, which can alter sCr levels [3,4,7]. Additionally, sCr levels do not rise significantly until approximately 75% of kidney function has been lost, limiting its effectiveness for the early detection of renal disease [7]. In other words, GFR can decline rapidly in the early stages of progressive kidney disease without a corresponding increase in sCr concentration. On the other hand, in the later stages of renal impairment, even a modest reduction in GFR can lead to a significant and rapid increase in sCr levels [10]. Nevertheless, sCr is generally preferred over blood urea nitrogen (BUN) as a renal function marker, given its reduced susceptibility to non-renal influences [10].

### 3.2. Symmetric Dimethylarginine (SDMA)

SDMA is released into the bloodstream during the degradation of proteins and is a product of the intranuclear methylation of L-arginine [9]. SDMA is predominantly eliminated through glomerular filtration and is not significantly influenced by tubular reabsorption or secretion. Therefore, it can serve as a reliable surrogate marker for GFR [9].

Unlike sCr, SDMA levels are not influenced by muscle mass, providing a more consistent reference range across diverse patient demographics. However, just like sCr, SDMA concentrations can be affected by factors such as hydration status, as well as biological and analytical variability [12,16,17]. A study by Couto et al. [18] found that Greyhound puppies exhibit higher SDMA values than would be expected for non-Greyhound puppies of the same age. Similarly, previous research has documented biological variability in serum SDMA concentrations among healthy geriatric cats [19].

The elevation of SDMA concentrations is directly proportional to the severity of renal dysfunction [17]. In a study conducted by Nabity et al. [20], dogs with X-linked hereditary nephropathy (XLHN) exhibited increasing levels of SDMA as the disease progressed, which correlated with rising sCr levels and declining GFR. However, SDMA levels may not be effective in distinguishing between AKD and CKD [21]. Furthermore, SDMA did not prove useful for predicting renal disease in dogs infected with *Leishmania infantum* [22] although it demonstrated efficacy in detecting glomerular toxicity in a rat model study [23]. In hyperthyroid cats, SDMA has shown a high specificity but poor sensitivity for the prediction of renal disease [24].

SDMA concentrations can increase with an average reduction of approximately 40% in GFR, allowing earlier detection of decreased kidney excretory function compared to sCr alone [16,25,26]. According to Relford et al. [26], veterinarians can diagnose kidney disease 2.4 times more frequently in cats and 2.0 times more frequently in dogs when utilizing SDMA rather than relying solely on sCr. In another study, McKenna et al. [6] demonstrated that SDMA can detect a decrease in GFR of less than 20% on average, which occurs earlier than changes in serum creatinine. Specifically, serum SDMA levels increase prior to sCr by an average of 9.8 months (with a range of 2.2 to 27 months) in dogs with CKD. Consequently, SDMA serves as a particularly valuable biomarker for the initial diagnosis of CKD in older patients or those with poor muscle mass [19,25].

A previous study found a weaker positive correlation between SDMA and sCr in dogs with AKD compared to those with CKD. This finding is somewhat unexpected, as one might anticipate that lower muscle mass in dogs with CKD would weaken the correlation between SDMA and creatinine [17]. In another study, it was reported that in dogs with CKD, SDMA concentrations correlate with sCr concentrations, with SDMA serving as an earlier indicator of renal dysfunction [21]. Furthermore, sCr concentrations were significantly higher in dogs with AKD compared to those with CKD, although SDMA concentrations were found to be similar between the two groups [21].

Together, SDMA and sCr may better identify populations at higher risk of kidney disease than when used alone [27].

A higher SDMA/creatinine ratio (>10) is associated with an increased risk of mortality in dogs and cats with CKD [21]. Therefore, SDMA shares some limitations with sCr in the detection of AKD, except that SDMA levels are not influenced by muscle mass [28].

### 3.3. Cystatin C (CysC)

Cystatin C (CysC) is a low-molecular-weight protein produced at a constant rate by all nucleated cells [29]. It is freely filtered across the glomerulus and subsequently reabsorbed by renal tubular cells. Consequently, serum concentrations of CysC increase as GFR decreases. However, urinary concentrations may increase following tubular injury [4,30].

It has been suggested that urinary CysC is more sensitive than other low-molecular-weight proteins in detecting renal dysfunction. Nevertheless, it is essential to measure total proteinuria, as significant proteinuria can inhibit tubular reabsorption of CysC [31,32].

According to Davis et al. [33], serum CysC serves as a marker of glomerular filtration rather than tubular injury. In this context, several studies [7,29,34] have indicated its effectiveness, demonstrating an apparent advantage over creatinine in early detection of renal impairment.

Similar findings have been reported in feline studies, as noted by Póswiatowska et al. [32]. In a recent study, Paes et al. [29] found that in critically ill dogs, measurement of CysC exhibited superior capability in identifying patients with AKD compared to the IRIS classification and sCr levels. However, other studies have reported poor correlation of serum CysC concentrations with GFR, including findings that serum CysC could not differentiate between cats with CKD and healthy controls [35,36].

The variability of this biomarker appears to be a subject of controversy according to the literature. A study by Braun et al. [37] demonstrated that plasma CysC levels vary with age, being lower in adult dogs and lower in dogs with a body weight of less than 15 kg. In dogs, there seem to be variations in CysC levels [38,39]. However, some other studies have failed to find a correlation between serum CysC and age or weight or with dietary factors [40]. In contrast, no biological variation in serum CysC levels has been observed in cats [32,41].

CysC levels appear to increase in patients with various non-renal diseases [7,42]. In dogs with leishmaniosis, this marker may be useful for monitoring the progression of renal disease [43,44]; however, urinary CysC does not effectively identify dogs with early kidney disease [45]. In hyperthyroid cats, serum CysC concentrations are elevated regardless of renal function [41]. Conversely, this does not occur in cats with diabetes mellitus [35,46] or in cats with feline immunodeficiency virus infection. Overall, serum or urinary CysC do not appear to be reliable biomarkers for the detection of early CKD in cats [47,48].

Thus, CysC is a superior screening test for detecting early renal impairment, while serum sCr concentration may serve as a more sensitive endogenous indicator of temporal changes in GFR once renal dysfunction has been established [42]. However, according to Pagitz et al. [49], the biological variability of CysC limits its utility as a superior marker compared to creatinine for detecting decreased GFR.

## 4. Markers of Glomerular Damage and Dysfunction

### 4.1. Albumin (Alb)

Albumin is the predominant serum protein in both dogs and cats, playing a crucial role in various physiological functions. It is synthesized by the liver and serves as a carrier protein, which is essential for maintaining oncotic pressure [11,48]. Under normal conditions, albumin is not present in significant quantities in the glomerular filtrate due to its relatively large molecular size and the selective permeability of the glomerular filtration barrier [11,48]. However, small amounts of albumin may pass through the glomerulus, but these are reabsorbed almost entirely by the proximal tubular cells [48]. The presence of elevated levels of albumin in urine can indicate a disruption in this filtration and reabsorption process. Albuminuria of glomerular origin is usually of greater magnitude [11,50].

Albuminuria has been shown to have a negative association with survival in both cats and dogs [51,52,53,54].

The measurement of albuminuria has been proposed as a screening test for early renal damage in dogs that are predisposed to or suspected of having renal disease [55]. However, albuminuria is not specific to renal diseases, as it can also be elevated in the presence of different infectious, neoplastic, or metabolic conditions, as well as cardiovascular diseases [53,55,56,57,58,59,60,61].

### 4.2. Immunoglobulins (IgG, IgM, and IgA)

Immunoglobulins G (IgG), M (IgM), and A (IgA) are serum proteins that are typically too large to pass through the glomerular filtration barrier in a healthy kidney. When the integrity of this barrier is compromised, these immunoglobulins can enter the urinary filtrate [8,11]. Dogs with renal diseases associated with conditions such as babesiosis [62], leishmaniasis [63], leptospirosis [64], hypercortisolism [65] poisoning [63], X-linked hereditary nephropathy (XLHN) [20], and pyometra [66] have shown an increase in urinary IgG levels.

Moreover, elevated levels of both IgM and IgG have been significantly associated with decreased survival time in dogs diagnosed with CKD [14].

## 5. Markers of Tubular Damage/Dysfunction

### 5.1. Cystatin B (CysB) and Clusterin (Clust)

Cystatin B (CysB) is an intracellular protein that is widely distributed in various cell types throughout the body and is typically not present in large concentrations in the bloodstream [67]. Therefore, the detection of CystB in serum or urine is indicative of tubular cellular damage, specifically resulting from the rupture and death of cells due to epithelial necrosis [28,68].

Studies indicate that CysB may serve as an earlier biomarker than creatinine for detecting AKD [68]. However, some research has shown a clear distinction between healthy patients and those diagnosed with AKD [48].

Urinary tract infections (UTIs) do not appear to influence CysB levels [68]. Segev et al. [67] presented CysB as a promising biomarker for the detection of active intrarenal injury in dogs with CKD and suggested it may serve as a potential surrogate marker for the rate of disease progression. Gordin et al. [28] and Chen et al. [69] have demonstrated that CysB not only provides insights into the severity of tubular injury but also helps in predicting outcomes and mortality following acute kidney injury in dogs and cats, respectively.

The ubiquitous nature of CysB does not allow the definitive conclusion that its presence in urine is only due to damaged renal tubular epithelial cells [28,67]. Moreover, its metabolism is not fully understood; so, it is possible that excretion from other organs contributes to the urinary concentrations observed [28,70].

Clusterin (Clust) is a protein expressed in numerous tissues, including kidney tissue [68]. Notably, the glycosylation patterns of kidney-specific Clust differ from those of the plasma isoform, enabling the measurement of kidney-specific Clust. Its detection in urine serves as an indicator of kidney injury affecting both proximal and distal tubules [28].

Furthermore, levels of urinary Clust have been observed to decrease upon recovery from kidney injury [68]. Consequently, urinary Clust may serve as a promising biomarker for the diagnosis and prognosis of AKD in dogs [28].

In response to renal damage, urinary Clust levels increase, whereas CysB, an intracellular protein, is released into the urine upon tubular cell injury [70]. These biomarkers have demonstrated sensitivity in detecting snake venom and gentamicin-induced renal proximal tubular injury prior to the elevation of sCr levels [28,68,70].

In dogs undergoing cardiac surgery with cardiopulmonary bypass, CysB levels were found to be elevated on the day of surgery, while urinary Clust levels increased by the second postoperative day in dogs with AKI and likely subclinical AKI [71]. Additionally, Le Sueur et al. [72] demonstrated that dogs infected with *Ehrlichia canis* exhibited early and higher concentrations of uCysB and urinary Clust, while SDMA and sCr remained unchanged.

Conversely, dogs with AKD classified as a lower IRIS grade did not exhibit significantly lower levels of urinary Clust or CysB compared to dogs with higher IRIS AKI grades. One possible explanation for this finding is that the IRIS grading system assesses kidney function, while urinary Clust and CysB serve as markers of structural damage. Functional and structural changes in the kidneys may not always correlate in a direct manner [28].

Currently, the scientific literature regarding the clinical utility of these biomarkers in cats is insufficient to draw definitive conclusions.

### 5.2. Neutrophil Gelatinase-Associated Lipocalin (NGAL) and Kidney Injury Molecule-1 (KIM-1)

Neutrophil gelatinase-associated lipocalin (NGAL) is an intracellular protein of hepatocytes, neutrophil granules, and epithelial cells, including renal tubular epithelial cells [73]. NGAL passes freely through the glomerular filtration barrier and is almost completely reabsorbed by the proximal tubules in a healthy kidney [8]. In the event of renal epithelial injury, the reabsorption of NGAL in the proximal tubules is impaired, while there is an upregulation of NGAL synthesis due to damage to tubular epithelial cells. This leads to an increase in plasma NGAL levels [8,74]. Moreover, glomerular damage that results in urinary protein overload can further hinder the tubular reabsorption of NGAL, while simultaneously increasing its production and release [8,75].

In dogs, elevations in urinary NGAL occur prior to increases in sCr levels in both AKD and CKD [12,20,74]. Research has demonstrated that uNGAL concentrations are significantly higher in dogs with AKI compared to those with CKD or UTIs [20,73,76,77]. Furthermore, urinary NGAL may be a useful biomarker for predicting the severity and risk of progression in dogs with CKD [77,78,79]. According to Kongtasai et al. [48], uNGAL levels may correlate more closely with the severity of azotemia or renal impairment rather than the specific type or progression of kidney disease. Notably, serum NGAL has been found to be less sensitive than urinary NGAL in dogs, including those with experimental leishmaniasis [80] and parvovirus-induced AKD [81].

Urinary NGAL is particularly noted for its utility in identifying various forms of kidney injury, including gentamicin-induced tubular damage [82,83,84], tenofovir disoproxil fumarate-related injury [85], heatstroke-induced kidney injury [86], envenomation-related tubular injury [70], ischemia/reperfusion injury [33], X-linked hereditary nephropathy [20], and post-surgical kidney injury [87].

Furthermore, this marker has been shown to be a valuable predictor of renal damage in animals suffering from myxomatous mitral valve disease (MMVD). The renal tubular damage was measured by increased urinary NGAL even in the absence of azotemia, and it increases with the severity of MMVD [88].

It is important to note that urinary NGAL levels can be influenced by white blood cell counts and the presence of pyuria in canine urine samples. Moreover, the presence of the urinary NGAL monomer seems to be correlated with renal injury, whereas the presence of dimeric urinary NGAL appears to be involved in pyuria and UTI [89]. According to Segev et al. [86], NGAL is one of the earliest and most robustly induced proteins observed in both humans and animals with AKD. However, it is worth mentioning that NGAL levels in both serum and urine are not considered reliable biomarkers for renal dysfunction in cats [90].

Kidney injury molecule-1 (KIM-1) is a transmembrane protein that is normally expressed in healthy proximal convoluted tubular cells and is shed into the urine at low concentrations [30]. Following injury to the proximal tubules, elevated levels of KIM-1 can be detected in both urine and the bloodstream [73]. KIM-1 is widely utilized in histopathology to identify renal tubular injury [91].

However, the clinical relevance of urinary KIM-1 has been reported variably in veterinary literature. This biomarker has demonstrated significant clinical utility in the early diagnosis of AKI, particularly in non-azotemic stages [92]. Additionally, urinary KIM-1 appears to be a useful predictor of tubular injury associated with various conditions, including venom exposure [93], leptospirosis [94], and babesiosis [95].

In contrast, several studies have reported that urinary KIM-1 is less reliable for detecting tubular injuries induced by cisplatin [96] and gentamicin [97,98]. Additionally, Bland [99] demonstrated that uKIM-1 may serve as a potentially useful indicator of proximal tubular injury in cats, but the reports on this species are lacking.

In dogs diagnosed with leptospirosis or babesiosis, NGAL and KIM-1 serve as early indicators of renal damage. These biomarkers are particularly valuable in cases of AKI that present without azotemia [95]. Additionally, the elevation of their levels in plasma serves as an early indicator of gentamicin-induced AKI in dogs [100]. Plasma NGAL and KIM-1 are effective in identifying stages of CKD and stratifying risk groups. These biomarkers demonstrate superior diagnostic accuracy compared to traditional indicators, such as sCr [73].

### 5.3. Retinol Binding Protein (RBP)

RBP is a low-molecular-weight protein synthesized by the liver that functions as a transport molecule for retinol in plasma. RBP can freely pass through the glomerular filtration barrier and is typically reabsorbed by the renal tubules, which allows it to serve as a marker for renal tubular function [4]. In healthy dogs, only minimal amounts of RBP should be excreted in the urine. When tubular damage occurs, there is a decrease in the reabsorption of RBP, leading to an increased loss of RBP into the urine. Additionally, glomerular damage may contribute to elevated urinary RBP levels [8]. Research has indicated that urinary RBP concentrations correlate with serum creatinine levels and GFR, suggesting that RBP may serve as a sensitive marker for AKI [12].

Increased concentrations of urinary RBP have been observed in dogs with CKD [101,102], urolithiasis [101], and hereditary nephropathy [20] compared to healthy dogs. Elevated levels of this biomarker are also noted in untreated hyperadrenocorticism [65] and in cats with untreated hyperthyroidism [30]. The measurement of urinary RBP may have clinical utility for the early detection and monitoring of CKD in dogs, as it has demonstrated progressive increases in values relative to other markers of renal function [20].

RBP appears to be less influenced by the magnitude of proteinuria compared to other evaluated urinary biomarkers [20]. However, urinary RBP levels were found to be higher in proteinuric dogs than in azotemic, non-proteinuric dogs, and no significant association was observed between urinary RBP levels and decreased GFR [52].

In cats with hyperthyroidism, urinary RBP is not indicated for the monitoring of renal function [103,104]. Furthermore, RBP exhibits considerable inter-individual variation in this species, suggesting that it may not be a suitable marker for renal tubular injury in felines [10].

Although RBP is a promising candidate as a urinary biomarker for tubular dysfunction in dogs, further studies are necessary to evaluate its utility in the diagnosis of AKI in canines [105].

**Figure 2:**
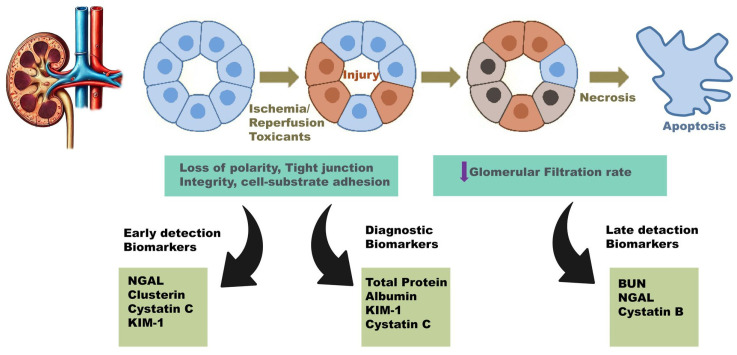
Kidney injury biomarkers (early markers and late markers). NGAL (neutrophil gelatinase-associated lipocalin); KIM-1 (kidney injury molecule-1); BUN (blood urea nitrogen).

In the medicine of companion animals, it has been demonstrated that epithelial damage occurs in animals with AKI before any increase in functional markers, as well as in animals with apparently stable CKD. The degree of epithelial damage is also associated with disease progression and survival, but more studies are needed to prove it [12]. Figure 2 aims to elucidate early renal injury biomarkers, which occur following initial epithelial damage, and those that arise after cellular apoptosis and necrosis, appearing later. These may potentially serve as markers for early diagnosis, or their detection may enable the evaluation of renal lesion prognosis. With these possibilities, a clearer distinction between CKD and AKD could be achieved [12]. Table 2 shows a summary of biomarkers showing the advantages and disadvantages of each one. This table also has the information about its relevance to AKD or AKD diagnosis.

Renal disease is a significant concern in small animal clinics, and the elevated number of reports demonstrates its importance. The mortality rate associated with renal disease is notably high, primarily due to the failure to diagnose early damage to the kidneys in these species. This is partly because the kidneys possess a substantial functional reserve, allowing normal GFR even when a considerable number of nephrons are damaged [4]. Traditionally, sCr, and more recently SDMA, have been widely utilized for the diagnosis and monitoring of kidney disease [5]. However, both biomarkers lack sufficient sensitivity and specificity for the early diagnosis of impaired kidney function.

Table 3 summarizes distinct studies that made a comparison between different biomarkers for a specific cause of kidney lesion.

The journey of a biomarker from the laboratory bench to the clinic is long and complex. Numerous biomarkers are discovered and reported regularly, yet only a small fraction reach clinical application. Many requirements and criteria must be met for a biomarker to be adopted in clinical practice [68]. Ideally, a biomarker should possess as many of the characteristics listed in Table 1 as possible. According to Obert et al. [107], biomarkers such as Kim-1, NGAL, and CysC have been utilized in veterinary diagnostics. However, these assays are not universally available in general veterinary clinics. Further research is warranted to validate and implement additional biomarkers into routine veterinary diagnostic practice.

Table 2 demonstrates that all biomarkers possess both advantages and disadvantages, and none of them exhibit the majority of the characteristics listed in Table 1. We aimed to identify sensitive and specific reference intervals for each marker; however, these intervals varied across studies and species, and a definitive ratio could not be established. These findings are expectable once renal disease is not a single pathological entity but rather the common outcome of diverse pathological processes that can occur concurrently. Consequently, it is improbable that a single biomarker will capture all relevant processes. Instead, biomarker panels or disease-specific biomarkers may offer improved prognostic utility for CKD.

Despite significant advancements in this field, there remain notable controversies regarding the interpretation and standardization of biomarker applications across different species (Table 2). It is evident that accepted reference ranges and cut-off values for biomarkers have not yet been established; this is primarily due to a lack of standardized analytical methods and large-scale studies [113]. Most biomarkers do not have standardized reference ranges. For instance, serum CysC, RBP, GAL, and IgG exhibit considerable intra-individual variation in healthy dogs. This variability can complicate their interpretation, although Liu et al. [113] noted that serum CysC demonstrated the lowest variation among these markers. Additionally, there are limitations regarding the targeted and quantitative evaluation of biomarker performance in specific clinical settings. The development and validation of reliable assay methods remain insufficient [4,5].

As a result of these findings, research has focused on grouping biomarkers by function, attempting to establish a pattern between early and late markers (Figure 2). The cells that constitute the framework of the renal tubules contain various enzymes in different parts of their structure. In the event of renal injury, their urinary activity will increase above the reference interval, allowing the identification of injury or the evaluation of the degree of renal lesion, such as extravasation or renal tubular necrosis [114]. NGAL, CLust, CysC, Kim-1, and Alb can be detected early, prior to the establishment of irreversible damage and the impairment of renal function, which subsequently results in increased creatinine and urea concentrations.

Other studies have aimed to correlate biomarker elevation with the presence of AKD and CKD. The use of specific biomarkers to identify ongoing kidney injury enhances the differentiation between AKD and CKD in human medicine and enables early detection of kidney damage [21]. Similarly, in veterinary medicine, the research has aimed to categorize biomarkers into AKI or CKD biomarkers. In this review, Table 2 also attempts to categorize them according to scientific findings. However, despite numerous researchers, discrepancies exist across various studies regarding the optimal biomarker to identify the different phases of renal disease. For instance, in dogs, NGAL has been considered a CKD biomarker in some studies [8,20,79], while in others [77,82,108] it was more significantly expressed in animals with AKI. Furthermore, discrepancies have been observed regarding the timing of biomarker release relative to epithelial injury. For instance, Cheon et al. [115] classify CysC as an early biomarker, whereas Vaidya et al. [116] consider it a late biomarker. A standard for their occurrence appears to be lacking. Certain biomarkers, such as KIM-1, Clust, and Cyst B, appear to be more closely associated with AKI in animals. Conversely, SDMA and CysC appear to be more closely associated with CKD [12].

There are other studies and analyses [68]. Table 3 presents several studies that investigate the relationship between various forms of kidney damage and elevated biomarker levels in blood and urine. This compilation aims to correlate specific biomarkers with particular kidney diseases. However, these studies exhibit limitations that complicate biomarker interpretation and validation for clinical use. Notably, the sample sizes in many of these studies are relatively small. [68] On the other hand, for a biomarker to have a significant diagnostic value, the differences between healthy animals and those with kidney disease must be substantial. However, neither of the studies reviewed demonstrate this level of distinction. The majority of the studies present in Table 2 did not have a control group. The complexities of kidney disease, along with the concurrent damage to other organs often observed in such cases, suggest that reliance on a single biomarker may be insufficient. Instead, a panel of biomarkers, each contributing unique and specific information, may be necessary [4,5]. Furthermore, research into multiple diagnostic approaches is essential to address this limitation [73]. In the majority of the studies in Table 2, the physiologic condition is not mentioned. The gender, the body condition, the type of nutrition, and the concurrent diseases were not clearly defined for all animals. Therefore, these results may vary with these aspects. Most existing studies (Table 2) use serum creatinine as an indirect marker of GFR, which is used to compare the performance of biomarkers, but ideally, renal clearance methods should be used Clique ou toque aqui para introduzir texto [10]. All these limitations contribute to the studies that exist to date and do not support the clinical use of these biomarkers. Another limitation is that different studies utilized different assay platforms for measuring renal biomarkers, and these findings cannot be generalized to other assay platforms. It is necessary to standardize the same biomarker to replicate it in future studies.

Studies have highlighted discrepancies in the efficacy of biomarkers between cats and dogs, raising questions about their universality and necessitating tailored approaches that consider each species’ unique physiological characteristics. While there is a substantial body of research focused on renal biomarkers in dogs, studies pertaining to these biomarkers in cats are relatively limited. Furthermore, it appears that the effectiveness of biomarkers in cats may be lower compared to that in dogs. These findings suggest that diagnosing renal issues in cats may be more challenging, which could discourage further research due to the perceived difficulties in obtaining reliable diagnostic tests. However, given that felines are more susceptible to renal diseases, there is an urgent need for increased investigation in this species.

It is important to note that, independently of the biomarker, the clinicians should interpret it in conjunction with the patient’s physical examination findings, especially for sCr and SDMA [8,20,21,77,79,82,108,115,116]. Additionally, it is essential that only urine samples exhibiting renal proteinuria—and not samples containing active sediments (e.g., hematuria, pyuria, or bacteriuria)—be utilized for the accurate interpretation of urinary biomarkers, particularly those indicating tubular damage [8]. Concurrent pathologies as well as medication should be part of the interpretation. This permits the elimination of or decrease in non-renal factors.

A longitudinal monitoring of these biomarkers is preferred for the determination of renal diseases compared with individual measurements [48].

In situations where it is necessary to access renal tubular function, it is crucial to utilize biomarkers that are specifically produced by the renal tubules or possess distinct renal tubular forms that can be differentiated from their circulating counterparts [14].

In this review, we observe that for an identical disease, the results can vary significantly across studies (Table 2). For instance, in Gentamicin-induced renal tubular toxicity studies, the three authors have different results on the efficacy of the biomarkers. This variation is largely attributed to the use of different biomarkers in each study, which complicates comparisons and interpretations between them. However, it can be generally deduced that urinary biomarkers of proteinuria are among the most useful analytes for the detection of glomerulonephritis [117]. And in most of the studies, it is notable that urinary biomarkers have a higher correlation with the presence or severity of kidney injury compared to serum or plasma biomarkers [30].

Several candidate substances, such as NGAL, KIM-1, and CysC, have been proposed as markers for the early stages of AKI and CKD, but their clinical utility is not well established. Furthermore, these biomarkers are not readily available to clinicians, and their associated costs may not be practical for widespread use. There is an ongoing debate regarding the best practices for integrating these biomarkers into routine clinical assessments. Despite the fact that most clinicians base their diagnostic approach on the most used biomarkers (such as sCK or SDMA), some published guidelines already mention the practical advantages and disadvantages of some advanced approaches and emerging biomarkers; for example, the International Society of Feline Medicine (ISFM) points out the importance of considering wider and more diverse approaches to some patients [117].

It is hardly possible to point to a single biomarker as an ideal substitute for creatinine. Despite its known limitations, creatinine remains a useful and practical marker of kidney disease. It is expected that in the future a panel of biomarkers, along with creatinine, will be used, rather than a single test, in order to obtain a better assessment of kidney function and to diagnose diseases as early as possible. A panel of biomarkers may offer a more nuanced understanding of renal health in companion animals, ultimately leading to improved diagnostic and therapeutic strategies in veterinary nephrology. Future applications may incorporate a personalized medicine approach, such as targeting patients undergoing therapy for renal fibrosis prevention or identifying AKD patients with preserved kidney function who are at risk of CKD progression. We anticipate that in the future, a panel of biomarkers will be commercialized to provide a comprehensive assessment and categorization of renal disease.

## 6. Conclusions

Kidney disease, particularly CKD, represents a significant health challenge in companion animals. Understanding the similarities and differences in clinical signs and biomarkers across species can provide insights into the diagnosis and management of these conditions. No single biomarker fulfills all diagnostic criteria effectively (Table 1).

Despite the progress made, the current body of knowledge regarding the use of renal biomarkers in veterinary medicine is still limited, necessitating further research to validate their efficacy and applicability. Each biomarker presents advantages and disadvantages, indicating that a multi-faceted approach may be required to develop a comprehensive panel of biomarkers that can provide the necessary information for accurate diagnosis and treatment strategies in veterinary medicine. Therefore, continuous research in this area of research is essential to enhance diagnostic capabilities and improve the management of renal diseases in companion animals.

## Figures and Tables

**Table 1:** Desired characteristics of a renal biomarker.

Characteristic	Reason	References
Detectable in urine and/or plasma	It can be assessed routinely and serves as an indicator of kidney function.	[5]
Unique and specific to the kidney	Should reflect specific kidney damage very early.	[4,13]
Provides insights into the etiology and location of the injury	It affects the glomeruli or tubules, or it should be prerenal, renal, or postrenal.	[4,13]
Reflects the severity and potential for recovery	Indicate kidney injury or repair processes and predict the likelihood of recovery.	[13]
Increases rapidly and reliably in response to kidney disease	The absence of a biomarker may predict resolution of the active phase.	[5]
Chemically stable	Does not interfere with drugs and should be stable over time and across different temperatures and pH levels.	[13]

**Table 2:** Overview of renal biomarkers in cats and dogs.

Biomarker	Samples	Species	Advantages	Disadvantages	CKD, AKD, or Both	References
Cr	Serum	Cats and dogs	Widely available. Inexpensive. Familiar assay. Most accurate in steady state GFR.	Non-linear relationship with GFR. Proportional to patient muscle mass. Influenced by pre- and post-renal azotemia and hydration status. Higher creatinine levels in breeds with increased muscle mass.	Both	[10,14]
SDMA	Serum	Cat and dogs	Increases progressively with increased renal impairment and progressive nephron loss in animals and humans with CKD.	Intra-individual and analytical variations are higher than those of serum creatinine. May be influenced by diseases such as diabetes mellitus, neoplasia (lymphoma), and nephrolithiasis.	CKD	[10,14,106]
Cyst C	Serum Urine	Cats and dogs	Good marker of GFR in early stages of renal disease. Demonstrated utility in human clinics and in animals.	Questionable effects of age and weight in dogs. Not consistently shown to be superior to creatinine as a marker of GFR. Diabetes in cats can influence the results.	AKD: Dogs CKD: Dogs and cats	[10,14,107]
Alb	Urine	Cats and dogs	High specificity for renal injury.	Immunoassays can underestimate low-level injury due to resorption/excretion of variably sized non-immunoreactive albumin fragments	AKD	[107]
Igs	Urine	Dogs	Can be helpful in diagnosing and monitoring glomerulonephritis.	Hematuria, pyuria, bacteriuria, and treatment with hydrocortisone can influence the results. Requires specialized laboratory techniques.	Both	[14,107]
Cyst B	Urine Serum	Dogs	Particularly valuable for detecting acute and active injury to renal tubular epithelial cells in early stages.	While valuable for tubular injury, it may not be as sensitive for other forms of kidney disease.	AKD	[28,48]
Clust	Urine	Dogs	May be an early indicator of renal injury. Could provide insights into the severity and progression of kidney disease.	Clusterin is involved in multiple biological processes; so, its elevation may not always be specific to kidney disease. Variability in results depending on the method of testing.	AKD: Dogs	[14,72]
NGAL	Urine Serum	Dogs	Good at predicting the progression of AKI to CKD. In a toxicity case, NGAL increased significantly several days before creatinine.	Hematuria and pyuria may cause assay interference. Malignancy, inflammation, and infection may decrease specificity. AKI marker with a large dynamic range in many species.	AKD: Dogs CKD: Dogs	[77,82,108] [8,20,79]
Kim-1	Urine Serum	Cats and dogs	Can potentially detect kidney injury earlier than creatinine.	Current dog assays are problematic. Undetectable in healthy cats.	Both, but mostly with acute processes.	[48,107]
RBP	Urine	Cats and dogs	Useful for monitoring chronic disease due to progressive increases in later disease stages	Wide intra-individual variation in feline CKD and hyperthyroidism. The availability and standardization of RBP assays in veterinary medicine may be limited.	AKD and CKD: Dogs	[101,102]

**Table 3:** Comparative effectiveness of various biomarkers across different studies.

Cause of AKD/CKD	Conclusions	Sample Size	Limitations	Reference
X-linked hereditary nephropathy (XLHN)	All urinary biomarkers elevated prior to an increase in sCr, but typically after the onset of proteinuria. uRBP/c may serve as a promising noninvasive tool for the diagnosis and monitoring of tubular injury and dysfunction in dogs with this pathology	25 dogs with XLHN and 19 unaffected	The small sample size and the lack of a control group. The study did not evaluate the biomarkers in dogs with other forms of renal disease, which could limit the generalizability of the findings.	[20]
22 different toxicants	Kim-1, Clust, and ALB showed the highest performance for detecting renal tubular injury. ALB used to detect glomerular injury. NGAL was the most nonspecific biomarker.	22 rats	Inability to differentiate the cause of Clusterin increases. The damage localization was unclear.	[50]
Gentamicin	CysC was the most sensitive indicator of kidney injury in dogs.	8 dogs: 4 gentamicin group 4 control group.	The small size, the short duration of gentamicin administration (7 days), and the use of only male dogs.	[82]
	NGAL and Clust were the most sensitive biomarkers.	12 dogs	Small sample size. The findings may not be directly applicable to other dog breeds or species [82].	[83]
	SDMA was a more immediate biomarker for detecting gentamicin-induced toxicity compared to sCysC, BUN, and sCr.	80 rats	Only male rats were used in the study, and it is possible that the results would have been different if female rats had been used.	[109]
Headstrock	URBP and UNGAL were increased in all dogs with heatstroke.	20 dogs	Small sample size and the absence of a control group of healthy dogs. The variability in heatstroke severity, which could have influenced the biomarker results. The information about pre-existing renal disease is not included.	[86]
Tenofovir disoproxil fumarate	Kim-1 and Clust were the most sensitive. CystC, RBP, NGAL, and ALB showed improved sensitivity over BUN and SCr.	24 animals: 12 monkeys and 12 dogs.	Small sample size and limited time points: more frequent sampling might have provided a more detailed picture of the biomarker changes over time. Further research is needed to understand how these biomarkers relate to specific pathological processes.	[85]
*Babesia* spp.	Urinary ALB and IgG indicated glomerular damage. Elevated levels of KIM-1 and RBP suggested proximal tubular damage.	42 dogs naturally infected with Babesia canis and 14 healthy dogs.	The researchers had to merge some of their predefined groups, which resulted in a smaller sample size for the analysis of certain markers. The creatine cut-off might be questionable because it does not account for differences in gender and muscle mass among the dogs.	[110]
Familiar glomerulonephritis in Doberman dogs	Urinary IgG can serve as a marker for glomerular function. uRBP has been identified as a marker for proximal tubular dysfunction.	20 Doberman Pinschers	The study population consisted only of Doberman Pinschers, limiting the generalizability of the findings to other breeds. A direct comparison with healthy dogs would have strengthened the conclusions. The disease stage at the time of sampling could affect the biomarker levels. Limited number of biomarkers.	[111]
Ischemia–reperfusion (IR)	Only NGAL showed a significant increase following IR. In contrast, sCysC was not useful in identifying early AKD related to IR in dogs.	12 dogs	The study involved a relatively small number of dogs. The study was conducted in dogs, and the findings may not be directly applicable to other species. The study compared the performance of different assay platforms for measuring renal biomarkers. The findings may not be generalizable to other assay platforms.	[33]
Poisoning by the European adder (snake)	KIM-1, NGAL, and ALB were indicative of renal tubular injury 12–36 h after envenomation. The observation of elevated AKI biomarkers at 36 h post-envenomation suggests poor prognosis. SDMA exhibited limited diagnostic utility in this context.	20 dogs	Small sample size and the absence of a control group of healthy dogs. The severity of envenomation, which could have influenced the biomarker results. The study does not explicitly consider the pre-existing renal disease.	[70]
Amphoterinicin B	uClust was the most sensitive biomarker.	12 dogs	Small sample size, the absence of a control group of healthy dogs, and the individual variation in response to amphotericin B.	[84]
Progressive feline kidney disease	SDMA and KIM-1 were sensitive biomarkers for early diagnosis and indicated an improvement in kidney function and repair. Can be potentially effective follow-up tools.	86 cats: 68 were assigned to the diseased group and 18 to the treated group.	Further research is needed to examine the biological variability of UPC in cats with kidney diseases and overt renal proteinuria, as well as in cats with elevated UPC ratios.	[112]

## Data Availability

No new data were created or analyzed in this study. Data sharing is not applicable to this article.
